# Oral microbiome and systemic antineoplastics in cancer treatment: A systematic review

**DOI:** 10.4317/medoral.25121

**Published:** 2022-04-03

**Authors:** Manuel Eros Rodríguez-Fuentes, Mario Pérez-Sayáns, Luis Alberto Chauca-Bajaña, Gema Barbeito-Castiñeiras, María Luisa Pérez del Molino-Bernal, Rafael López-López

**Affiliations:** 1 Oral Medicine, Oral Surgery and Implantology Unit (MedOralRes), Faculty of Medicine and Dentistry, Universidade de Santiago de Compostela, A Coruña, Spain; 2 Health Research Institute of Santiago de Compostela (IDIS), A Coruña, Spain; 3 Periodontics and Implantology Oral Research Unit. College Dentistry. Universidad de Guayaquil, Ecuador; 4 Microbiology Unit, Complejo Hospitalario Universitario de Santiago de Compostela, A Coruña, Spain.; 5 Medical Oncology Unit, Complejo Hospitalario Universitario de Santiago de Compostela, A Coruña, Spain

## Abstract

**Background:**

Oral mucositis is one of the most common side effects in cancer patients receiving systemic antineoplastics. However, the underlying biological mechanisms leading to this condition are still unclear. For this reason, it has been hypothesised that systemic antineoplastics may cause an imbalance on the oral microbiota that subsequently triggers oral mucosa damage.

**Material and Methods:**

A systematic review was performed following the PRISMA protocol and the PICO question established was: patients diagnosed with cancer, who are candidates for receiving systemic antineoplastics (*P*=Patients), that undergo oral microbiome determinations (I=Intervention), before and after systemic antineoplastics administration (C=Comparison), to analyse changes in the oral microbiome composition (O=Outcome). The bibliographic search was carried out in PubMed and other scientific repositories.

**Results:**

Out of 166 obtained articles, only 5 met eligibility criteria. Acute myeloid leukaemia (AML) was the most frequent type of cancer (40 %) among the participants. Only one of the studies included a control group of healthy subjects. Heterogeneity in the protocols and approaches of the included studies hindered a detailed comparison of the outcomes. However, it was stated that a decrease in bacteria α diversity is often associated with oral mucositis. On the other hand, fungal diversity was not associated with oral mucositis although α diversity was lower at baseline on patients developing oral candidiasis.

**Conclusions:**

There is insufficient scientific evidence of oral microbiological changes in patients undergoing systemic antineoplastics. Further investigations ought to be carried out to identify microorganisms that might play a key role in the pathogenesis of oral mucosa damage in patients undergoing systemic antineoplastics.

** Key words:**Stomatitis, antineoplastic agents, neoplasms, microbiota, immunotherapy.

## Introduction

Cancer is not a single disease but a variety of more than a hundred different conditions triggered by an uncontrolled cell growth that can be originated in any tissue. These cells have the potential to disseminate to other parts of the body and invade surrounding tissues (1). In 2020, the International Agency for Research on Cancer estimated a worldwide cancer incidence of 19.3 million of new cases (2). Consequently, a considerable percentage of them may require the use of traditional chemotherapeutic agents alone or in combination with surgical resections or radiotherapy (3). Nevertheless, new targeted therapies are becoming essential in recent years showing extremely successful outcomes (4). These treatments often lead to toxicity in healthy tissues, being oral mucositis (OM) one of the most common side effects. It consists of oral mucosal damage, previously described as a five-phase process (5), with inflammation, erythema, atrophy and/or ulceration of the oral cavity lining (6). Its incidence in patients with solid tumours receiving chemotherapy is between 20-40%, while in patients undergoing hematopoietic stem cell transplantation is between 60-80% and even superior in patients undergoing radiotherapy (7). Acute localized pain in the affected area, inadequate nutrition/malnourishment and dehydration are the most frequently reported signs and symptoms associated with stomatitis. It can be such a limiting factor that may even influence on postponing the administration of new doses of anti-tumour treatment until oral health is recovered, requiring patients’ hospitalization or prolonging the stay of those who were initially hospitalized. Therefore, not only cancer progression is a major concern, but important economic repercussions derived from the undesired effects arisen (8). The World Health Organization (WHO) established a specific tool to facilitate and homogenize clinicians grading of oral mucositis, a 0 to 4 scale based on clinical parameters (9). Currently there is no sufficiently effective treatments or preventive strategies for the management of oral mucositis, which leads to the use of methods aimed at palliation of the symptoms. Thus, there is a need to characterize the interactions in the underpinning biological pathways, with the aim of seeking effective alternatives for prevention and/or treatment (8,10). Despite the known cytotoxic effect of antineoplastics on the mucosa cells because of their speed of replication (11), it has been proposed that oral microbiota may have a crucial effect on the development of stomatitis due to the close relationship between microorganisms and mucosal tissue (7,12).

Oral microflora is defined as the set of different germs that inhabit the oral cavity, mainly integrated by bacteria, but also by protozoa, fungi, and viruses (13). The relationship created between these microorganisms and the host is basically commensalism (14), providing a protective barrier (15). Therefore, an alteration in the balance of its components might generate modifications at different signalling pathways’ levels and, subsequently, triggering pathological processes on the host’s oral health (16,17). Most of the oral microbiome studies in cancer therapy-induced oral mucositis were performed on patients receiving radiotherapy (12,18). However, only a few studies have linked changes in the oral flora of patients undergoing chemotherapy to the development of more advanced degrees of oral mucositis (19). For this reason, we hypothesized that systemic antineoplastic treatments (such as chemotherapy, immunotherapy, or targeted therapies) may influence changes on the oral microbiome of patients and, subsequently, dysbiosis might be responsible for the development of oral mucositis. Herein, our proposal focused on performing a systematic review to analyse changes in the relative abundances, of taxonomic, phylum, class, order, family, genus, or species of the different microorganisms that constitute the oral biofilm. Secondary objectives aim to investigate if more pronounced imbalances on the oral microbiome would correlate to changes in the severity of the oral mucosa damage.

## Material and Methods

- Protocol design and registration

The protocol for this study was designed following the Preferred Reporting Items for Systematic Reviews and Meta-Analyses (PRISMA) guidelines (20) and later registered on PROSPERO (ID: CRD42021236167). To establish a well-defined research question, we used a Patient-Intervention-Comparison-Outcome (PICO) method (21): patients diagnosed with cancer who receive or are candidates for receiving systemic antineoplastics (*P*=Patient); any determinations of oral microbiome (I = intervention); before and after systemic antineoplastics administration and in healthy controls if available (C=Comparison); changes in microorganisms' relative abundances of oral flora or in oral microbiome composition (O=outcome).

- Sources of information and search strategy

A bibliographic search was carried out in various scientific electronic repositories: PubMed/MEDLINE, Web of Knowledge, Cochrane, Directory of Open Access Journals (DOAJ), Literatura Latinoamericana y del Caribe en Ciencias de la Salud (LILACS) and SciELO from inception to August 2021. Furthermore, a manual electronic search was performed on field-related journals’ websites. Potentially relevant articles that any of the authors were familiar with, as well as reference lists from the retrieved articles, were also comprehensively checked.

Our team agreed a search strategy defined by the following algorithm with the aim of being applied in MEDLINE: ("oral microbiome" OR "oral microbiota" OR "oral biofilm" OR "oral flora" OR "oral microflora") AND ("Antineoplastic agent" OR Chemotherapy OR Immunotherapy OR "targeted therapy" OR "Induction Chemotherapy" OR "Molecular Targeted Therapy") AND (stomatitis OR "oral mucositis" OR "oral ulcer"). In addition to the forementioned formula, we also applied the “human” filter. The syntax was specifically adapted for other databases’ search, being the main keywords “oral microbiome” and “cancer therapy”.

- Eligibility criteria

All references identified from computerized databases were manually retrieved and the studies were included if they met the following inclusion criteria: All original articles, case reports and case series, with no language limitations. Longitudinal studies in subjects ≥18 years old that analysed oral specimens obtained at least before and after systemic antineoplastic administration, were also eligible. Included articles must perform microorganisms’ sequencing. The exclusion criteria englobed: technical features such as the use of different methods for germ identification, the inclusion of either paediatric patients or subjects undergoing radiotherapy. Other bibliographic aspects led us to discard letters, congress abstracts, literature reviews, systematic reviews, doctoral thesis, and original *in vitro* and *in vivo* studies.

- Study selection and data extraction process

Two independent researchers (M.E.R.F. and L.A.C.B.) performed selection of the studies by reading the titles and excluding those that did not focus on our topic. Secondly, abstracts of the retrieved articles were assessed and selected those that met eligibility criteria. Then, both researchers proceeded to fully read the remaining articles and discarded the unselected ones appropriately. In case of discrepancies, a third researcher (M.P.S.) acted as a mediator to conclude if articles met eligibility criteria. Data extraction process was performed, creating a database with all the variables available on each article that were relevant to the study. Finally, the two researchers compared results to ensure they matched.

The following information was extracted from each selected study when available: first author, year of publication, sociodemographic characteristics of subjects (number of subjects, age, gender, smoking habits), clinical parameters (acid inhibitors use, inhalers use, oral health-related examinations, type of diagnosed cancer), received treatments (type of chemotherapeutic agent, doses, antibiotic use and type, antifungal use and type), specimens obtained (type of specimen, method of obtaining, time of specimen extraction, sequencing method, sequenced regions), microbiome-related variables (any taxonomic, phylum, class, order, family, genus and species-related information given, bacterial relative abundances, bacterial and fungal alpha/beta diversity, bacterial/fungal changes associated with other risk factors, bacterial-fungal interactions) and oral mucosa damage assessments (oral mucositis and oral candidiasis development, peak of oral mucositis development, World Health Organization Scores (22)).

- Evaluation of quality and risk of bias

A quality assessment was performed by the two researchers to evaluate the risk of bias. We classified the five incorporated articles as cohort studies even though most of them did not include a control group (23). Thus, the Newcastle-Ottawa Quality Assessment Scale (NOS) for cohort studies was the method used (24) to obtain scores. The quality assessment stablished a rating method where: an overall star score of ≥ 7 stars was defined as high quality, medium quality when it was between 4 – 6 stars and low quality when below 4 stars.

- Statistical analysis

The means of certain values were calculated using SPSS software (IBM Corp. Released 2020. IBM SPSS Statistics for Windows, Version 27.0. Armonk, NY: IBM CorpArmonk, NY: IBM Corp) when appropriate.

## Results

- Study selection

**Figure 1:**
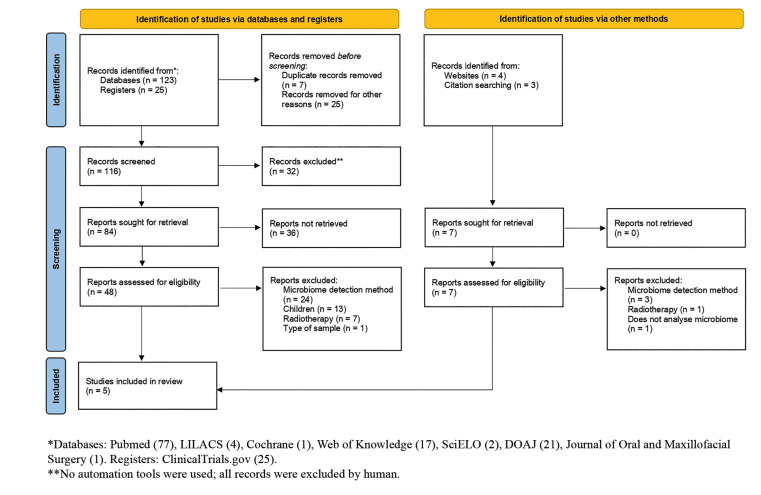
PRISMA 2020 Flow diagram for new systematic reviews.

A total of 155 references were retrieved from the initial search, out of which 150 were removed as detailed in Fig. 1. Subsequently, only 5 articles (25-29) met eligibility criteria to be included in our review.

- Quality and protocols details of the included studies

In Table 1 we summarize the quality assessment performed on the five articles that axis this review. According to the NOS, only one of them was classified as high quality, while the other 4 were medium quality. Comparability was only star-rated in one of the studies since the others did not include a healthy control group.

Among the selected articles, Hong *et al*. 2019 and Diaz *et al*. 2019 were conducted under the same protocol, which means that both investigations used data from the same population. Nevertheless, Diaz *et al*. 2019 excluded some patients because some specimens or other relevant clinical information were missed. Moreover, healthy controls were considered by Hong *et al*. 2019, but Diaz *et al*. 2019 did not analyse them. Similarly, Robinson *et al*. 2020 and Galloway-Peña *et al*. 2017 were articles derived from the same clinical study, however the most recent publication focused on extracting additional data that was not considered within the article initially issued.

- Main characteristics of the included articles

Our analysis showed in Table 2, reveals the main characteristics of the included articles. All these studies were observational and published between 2017 and 2020. Due to duplicities in the sample population among studies, it should be stated that 177 patients participated, after removing 51 (26) and 45 subjects (29).

**Table 1:**
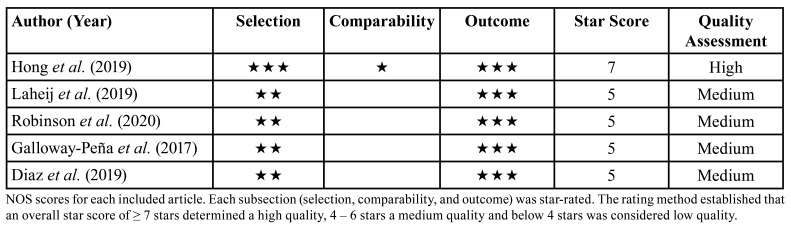
Quality Assessment through Newcastle Ottawa Scale of the included articles.

**Table 2:**
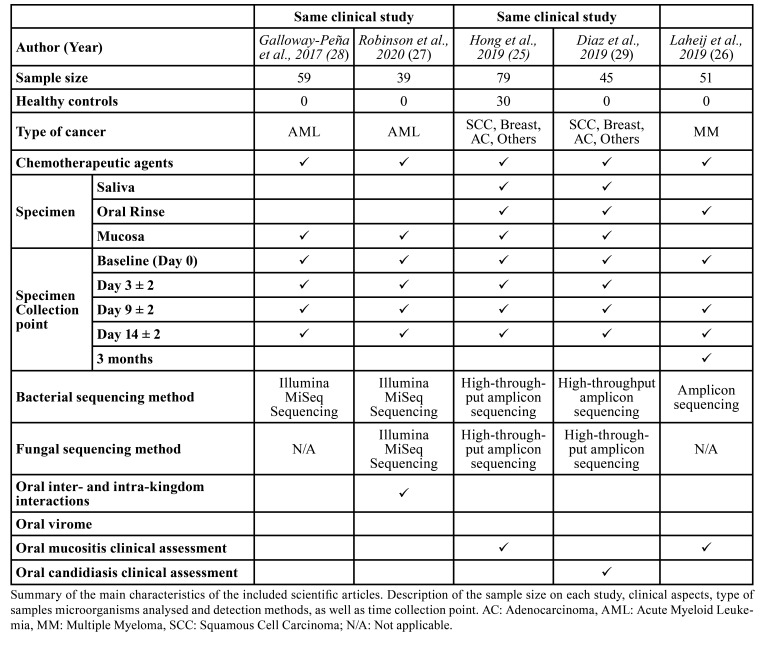
Summary of the main characteristics of the included scientific articles.

Having declared that, from this point onwards, we will consider articles as if they originated from independent research projects to avoid confusion.

A fair similarity of the sample size was noted among the included studies (39 – 59 cancer patients), regardless of healthy controls, which were exclusively included in one of them.

Unhealthy subjects mainly suffered from five different types of cancer, which were acute myeloid leukaemia, multiple myeloma, squamous cell carcinoma, breast cancer and adenocarcinoma. There were quite a wide variety of chemotherapy-based regimes administered to the patients in combination with adjuvants and immunotherapy.

The most frequent type of specimen collected in the studies was mucosa swab, followed by oral rinse while whole saliva was the least frequently used. Within all analysed studies, specimens were collected in a minimum of 4 different time points, 100% of them coincided in specimen collections at baseline (prior to treatment administration), day 9 ± 2 and day 14 ± 2. Only one of the studies differ from the rest, which collected a specimen in a follow-up visit 3 months after treatment initiation and did not obtain a specimen on day 3 ± 2 likewise the other 4 studies.

Microorganisms were detected through three different methods based on 16S rRNA sequencing. Two authors chose Illumina MiSeq sequencing, whereas other two preferred high-throughput amplicon sequencing (454 GS FLX) and only one of them used amplicon sequencing. Nevertheless, fungal sequencing was carried out in 3 studies, out of which 2 opted for high-throughput sequencing and 1 for Illumina MiSeq. Conversely, none of the incorporated studies to this review analysed oral virome of the enrolled individuals.

Regarding the clinical evaluations, 3 articles monitored oral mucosa damage. Two research teams graded it by examination, and registered stomatitis using other scales. Specifically, another article focused on oral candidiasis determination in chemotherapy-receiving patients.

- Quantitative data extracted

A mean of 49 subjects were included in each study, and the mean age was 56 years. An average of 50% of the participants were male and 74% received any type of antibiotic. Among the included cancer patients, acute myeloid leukaemia was the most frequent type of cancer (40%), followed by multiple myeloma (20%) and squamous cell carcinoma (17%), as shown in Supplement 1.

- Summary of oral microbiome-related outcomes

Galloway-Peña *et al*. 2017 (28) stated an association between the increase of three pathogenic-associated bacteria genera proportions and the α and β diversity transient variability, in the mucosa of patients receiving chemotherapy (shown in Table 3).

**Table 3:**
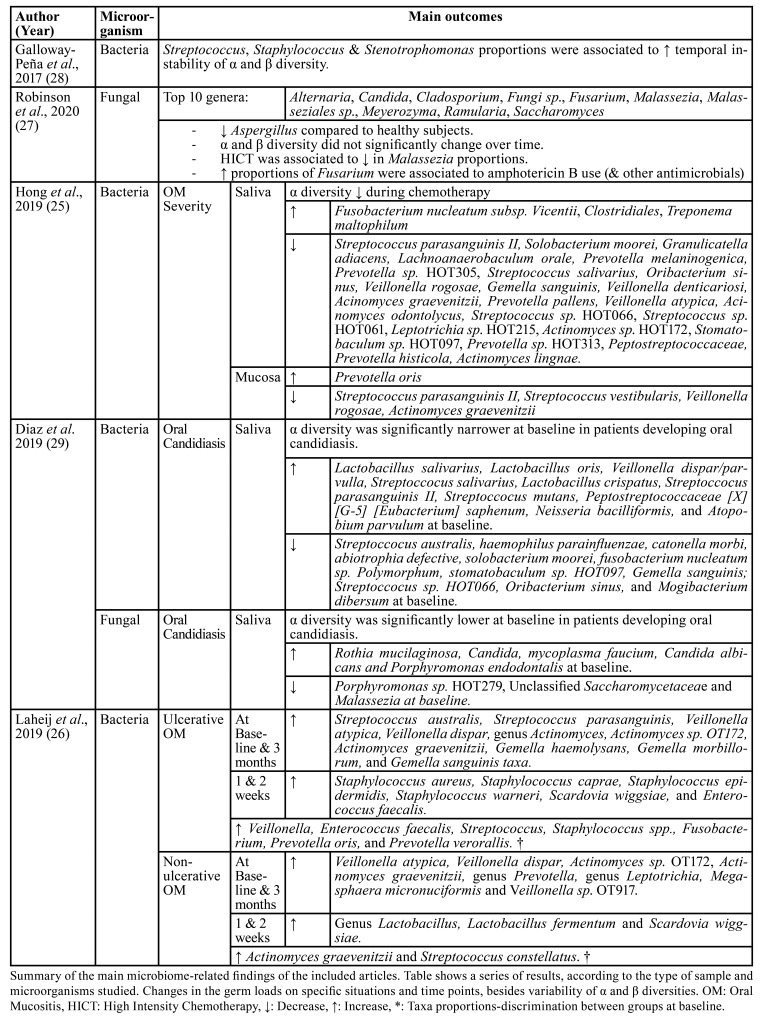
Summary of the main microbiome-related findings of the included articles.

Furthermore, the total days of antibiotic use had a similar effect on the oral microbiota fluctuations (28).

Next, Robinson *et al*. 2020 (27) displayed various oral mycobiome findings also detailed in Table 3. They did not state significant changes in α or β mycobiome diversity along time in patients receiving anticancer treatment. Whereas they revealed the 10 most abundant fungal genus in the oral cavity of these patients (Table 3). Among them, Malassezia, *Candida*, Saccharomyces, Fusarium, and Cladosporium turned out to be the top five, which matches findings in healthy individuals (27). Also, a series of intra- and inter-kingdom interactions were outlined as shown in Table 4.

Hong *et al*. 2019 (25) found that peak mucosa damage correlated to a lower salivary bacterial α diversity, overlapping the period where treatment is being administered. Specifically, they identified that the severity of oral mucositis was associated with disruptions in the oral bacteria loads. Increases and decreases in oral bacteria are detailed in Table 3, according to the type of specimen. Apart from that, they did not decipher any significant disturbances in oral salivary fungal diversity in relation to chemotherapeutic agent’s administration (25).

The investigation carried out by Diaz *et al*. 2019 (29), detected some components of the oral biofilm that might influence the development of oral candidiasis, described in Table 3. *Candida* communities were significantly higher at baseline in patients that later developed oral candidiasis, being *Candida albicans*, *Candida* Dubliniensis, and *Candida glabrata* the most abundant (29).

**Table 4:**
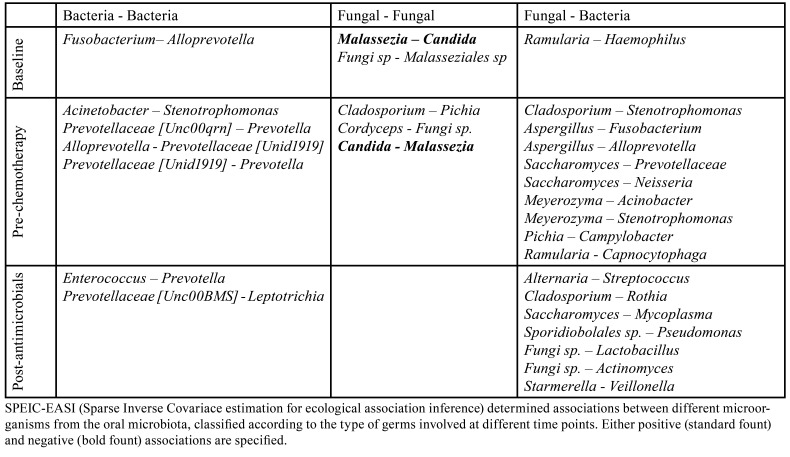
Summary of cross-domain association networks interactions described by Robinson *et al*. (2020).

On the other hand, Laheij *et al*. 2019 (26) structured their microbiome analyses dividing patients according to the development of ulcerative oral mucositis. They described a list of bacteria enrichments identified on each group at two different time points, as well as the main differences of augmented bacteria across the time. All these findings are detailed in Table 3.

## Discussion

Oral mucositis in cancer treatment is a major issue and oral microbiome might play a key role in this process (30). Herein, any findings in this field might be useful in the pursuit of novel treatments or preventive methods for this condition (31). It may seem that the study of microbiota is a current trend however, the reality is different in the specific case of oral damage in patients undergoing systemic antineoplastics. Even though a considerable volume of articles were retrieved after applying the search strategy for the bibliography search, the majority were not suiTable for inclusion. Many research articles were carried out using methods for microorganisms’ detection that have been proven imprecise (32). Moreover, a big volume of the retrieved literature included children, and evidence of age-related changes in the oral microbiome composition led us to exclude them (33). Similarly, a large proportion of the studies focused on oral microflora of cancer patients receiving radiotherapy, this is probably due to the high incidence of oral mucositis in this type of population (7). However, our interest from a medical oncology perspective conducted us to reject those, and include only articles where subjects underwent chemotherapeutic agents, immunotherapy, or targeted therapies.

All the analysed articles had quite homogenic groups of cancer patients, in terms of age and gender. Regarding other aspects, no clinical or microbiological consensus was noted across them, for this reason, meta-analysis was not performed. Interestingly, we did not find studies on oral microbiome that focused on patients receiving immunotherapy alone or targeted therapies. This is probably due to a low incidence of mucosa damage in these types of regimes compared to others (34).

In terms of type of specimen, oral rinse was found to be the most representative of the whole oral cavity microbiome, while mucosa swab specimens showed limited outcomes restricted to the specific areas that they were collected from (35,36). Diversity in the way results were expressed within the analysed studies, lead us to consider that the relative abundance of each germ type would be the most appropriate measurement for a quantitative analysis. Having this data from all the included articles would allow us to obtain better evidence.

The emerged systematic review is framed within the limits of the five included articles which generated restricted results. Based on the application of eligibility criteria, we noted a lack of consensus on the protocols used to study this topic. Although only 5 were included, the heterogeneity and diversity in the ways results were expressed, as well as the different approaches, hinders comparisons among them. The prime limitations of this review are concerning the different methodologies used on each of the included articles. Firstly, the sample size does not seem to be sufficient to obtain clear results, only one of the studies included healthy controls and there were also a wide variety of cancer types, mixing solid tumours and haematological cancers, as well as plenty of different chemotherapeutic agents. The types of specimens are also a negative point, using whole saliva that has been previously described to be less accurate and representative of the whole oral microbiome than oral rinse (36). The focus of these studies was to analyse the microbiological aspects, leaving on one side most of the clinical information, which may be very relevant for healthcare providers such as oral mucositis assessment and toxic habits. In terms of outcomes, the lack of quantitative results that express relative abundances of each microorganism proportions makes it impossible to find similarities and discrepancies that can generate overall conclusions and focus on specific microorganisms that may be directly involved in oral mucositis development. The variety of results found are confusing and their interpretation requires a rather arduous task of deciphering.

- Conclusion and future insights

The aim of this research was to characterize shifts in the proportions of oral microbiome components that arise because of systemic antineoplastics treatment. The results reveal insufficient scientific evidence of oral microbiological changes in patients undergoing systemic antineoplastics. Subsequently, we foresee an investigation niche focused on deciphering those imbalances as well as possible interactions between oral microbiome and mucosa. Outcomes would help us to find possible biomarkers that allow identifying patients who are susceptible of developing oral mucositis, and either use or design novel preventive strategies or treatments that reduce the severity of this adverse event. In summary, research with an appropriate number of adult cancer patients undergoing systemic antineoplastics-based regimes, besides healthy controls, should be the aim in future studies. Oral rinse should be considered the most representative specimen type of the whole oral cavity-microbiome and outcomes should be expressed as relative abundance proportions of each microorganism. The analysis should, at least, include bacteria and fungi if virus study is not feasible.
